# Towards a table-top microscope for nanoscale magnetic imaging using picosecond thermal gradients

**DOI:** 10.1038/ncomms9460

**Published:** 2015-09-30

**Authors:** J. M. Bartell, D. H. Ngai, Z. Leng, G. D. Fuchs

**Affiliations:** 1 School of Applied and Engineering Physics, Cornell University, Ithaca, New York 14853, USA

## Abstract

Research advancement in magnetoelectronics is challenged by the lack of a table-top magnetic measurement technique with the simultaneous temporal and spatial resolution necessary for characterizing magnetization dynamics in devices of interest, such as magnetic memory and spin torque oscillators. Although magneto-optical microscopy provides superb temporal resolution, its spatial resolution is fundamentally limited by optical diffraction. To address this challenge, we study heat rather than light as a vehicle to stroboscopically transduce a local magnetic moment into an electrical signal while retaining picosecond temporal resolution. Using this concept, we demonstrate spatiotemporal magnetic microscopy using the time-resolved anomalous Nernst effect (TRANE). Experimentally and with supporting numerical calculations, we find that TRANE microscopy has temporal resolution below 30 ps and spatial resolution determined by the area of thermal excitation. Based on these findings, we suggest a route to exceed the limits imposed by far-field optical diffraction.

## Abstract (web summary)

Table-top measurements of magnetic structures and dynamics in thin films are typically limited by temporal or spatial resolution. Here, the authors present a heat-based spatiotemporal magnetic microscopy method based on the anomalous Nernst effect, which may potentially overcome such limits.

## Graphical abstract

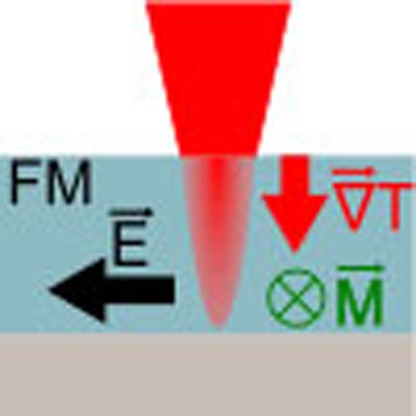

Recent advances in magnetoelectronics have demonstrated the potential for spin-based technology, including magnetic random access memory^1^,^2^, nanoscale microwave sources^3^,^4^ and ultra-low power signal transfer^5^. Magnetic microscopy has played a fundamental role in these areas through the illumination of magnetic behaviour such as domain wall motion^6^,^7^, magnetic switching^8^ and spin wave propagation^9^. Advanced microscopy techniques capable of examining local magnetization dynamics at length and timescales fundamental to spatiotemporal variations in magnetic systems^10^—typically 10–200 nm (refs 11, 12, 13, 14, 15) and 5–50 ps—would enable engineering advances and new scientific discoveries.

Currently, magneto-optical measurements are the only table-top approach to stroboscopically measure spatially varying magnetic dynamics. Unfortunately, the spatial resolution available to optical measurements is limited by diffraction to approximately *λ*/(2 NA), where *λ* is the wavelength of light and NA is the effective numerical aperture of the focusing optics. Therefore, optical techniques, including the time-resolved magneto-optical Kerr effect, have a diffraction-limited resolution of roughly 200 nm using blue light. One solution is to use radiation with a nanometre-scale wavelength, as in X-ray magnetic circular dichroism experiments, which provide spatial resolution down to 30 nm and time-domain resolution of <100 ps (ref. 16). Unfortunately, spatiotemporal X-ray magnetic circular dichroism requires synchrotron-based sources, which limits its widespread use.

To circumvent the spatial limitation imposed by optical diffraction, we propose a technique for magnetic spatiotemporal microscopy that uses the interaction between magnetization and heat, rather than light. Our method is based on the time-resolved anomalous Nernst effect (TRANE). The geometry for TRANE is depicted in Fig. 1a. The anomalous Nernst effect (ANE) is a magnetization-dependent, thermoelectric effect^17^,^18^,^19^ in which a gradient in the local temperature, *T*, transverse to the film's magnetic moment, generates an electric field, **E**_ANE_(**x**,*t*), given by ref. 20


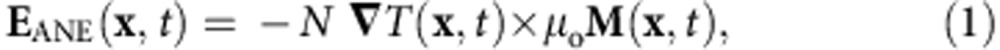


where *N* is the anomalous Nernst coefficient, *μ*_o_ is the permeability of free space, **∇***T*(**x**,*t*), is the thermal gradient and **M**(**x**,*t*) is the magnetic moment. Previous studies have demonstrated that by confining **∇***T*(**x**,*t*) to a micron-scale region in a thin-film ferromagnetic metal, an anomalous Nernst voltage is generated that is proportional to the local magnetic moment^21^,^22^. This has inspired proposals for applications that include microscopy and spectroscopy^21^,^22^,^23^,^24^. Up until this point, demonstrations and proposals have used thermal gradients that change slowly and heat a relatively large area of the sample under investigation. Here, we use a localized, pulsed thermal source with a short duty cycle from a focused laser to show **E**_ANE_ can be localized in both time and space to generate a TRANE signal. By synchronizing a magnetic excitation with thermal pulses, we use TRANE microscopy for stroboscopic measurement of magnetization dynamics.

## Results

### Magnetic measurement with pulsed thermal gradients

**Figure 1:**
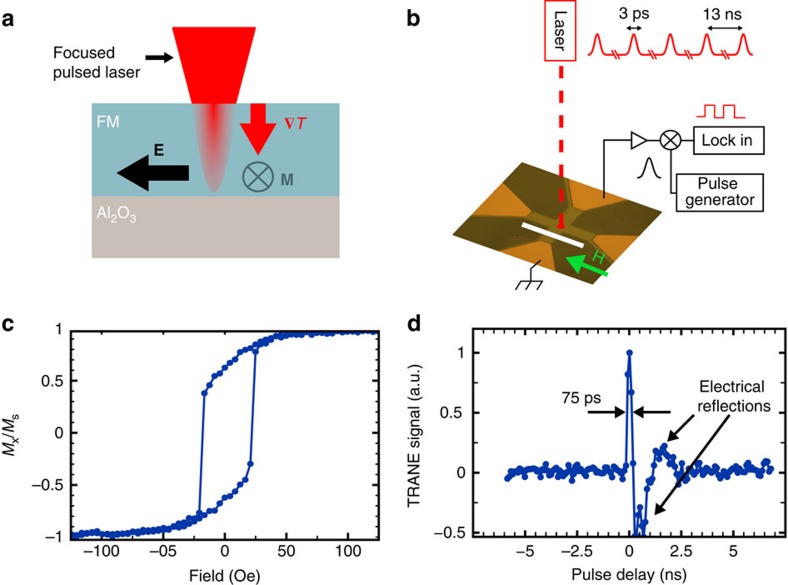
The TRANE for magnetic measurement. (**a**) Schematic diagram of the ANE in our measurements. A focused, pulsed laser creates a thermal gradient, **∇***T*, into the plane of a conducting ferromagnet (FM), with magnetic moment, **M**. The resulting electric field, **E**, leads to a voltage that we measure with electrical contacts. A thermally conductive, electrically insulating substrate (in this case sapphire) allows for efficient thermal sinking. (**b**) Schematic of the experimental set-up. A 792 nm pulsed laser is focused to a diffraction-limited spot on the magnetic film patterned on top of a sapphire substrate. Bonding pads enable detection of the ANE voltage pulse proportional to the perpendicular magnetic moment. The TRANE signal is measured with a lock-in amplifier after electrically mixing the ANE voltage pulse with a pulse from a pulse pattern generator. The white scale bar is 100 μm. (**c**) TRANE measurement of local hysteresis in the cobalt cross, normalized by the value at saturation, *M*_*s*_. Error bars (obscured by the point markers) are the s.d. of the data at saturation. (**d**) Signal from mixing the ANE voltage pulse with a 75 ps electrical reference pulse. We observe that the width of the initial peak in the mixed signal is 75 ps, indicating that the ANE pulse is <75 ps. The other, broader features are electrical reflections due to the impedance mismatch of the device and the 50 Ω transmission line.

Figure 1b shows a schematic of the measurement set-up. We focus a pulsed laser to generate a short-lived, local temperature gradient for each optical pulse, thus creating a corresponding voltage pulse, *V*_ANE_. A time-domain homodyne technique is used to convert the *V*_ANE_ pulses to a d.c. voltage, *V*_TRANE_, which is measured with a lock-in amplifier. *V*_TRANE_ is proportional to the stroboscopically sampled local magnetization as described in Supplementary Note 1. In this experiment, we used 792 nm optical pulses with a fluence of 2.3 mJ cm^−2^, which creates vertical thermal gradients of 4.0 × 10^8^ K m^−1^ at the surface, corresponding to a maximum temperature increase of 60 K (temperature measurement details in Supplementary Note 2 and Supplementary Figs 1–5 and Supplementary Table 1). The first structure we studied was a 30 nm cobalt film patterned into a 18 μm-wide cross-structure, shown in Fig. 1b. Figure 1c shows a hysteresis curve of this sample measured using TRANE, which demonstrates the proportionality between *V*_TRANE_ we measure and the local magnetic moment.

To test that the optically generated thermal gradients are short-lived, we perform a time-resolved measurement of the *V*_ANE_ pulses by electrically mixing them with 75 ps electrical reference pulses from a pulse generator (Fig. 1b). Figure 1d shows our measurement as a function of the delay between the reference pulses and the *V*_ANE_ pulses. We find that the resulting signal has a roughly 75 ps full-width at half maximum (FWHM). These data can be understood as the temporal convolution of the *V*_ANE_ pulses with the reference electrical mixing pulses. Since the convolved signal width is similar to the reference pulse width, the *V*_ANE_ pulses must be shorter than 75 ps (details in Supplementary Note 3 and Supplementary Fig. 6). As we show with subsequent magnetic resonance experiments, thermal gradients produced in our microscope are actually much shorter lived.

We now discuss the signal sensitivity of TRANE microscopy, which is dependent on several factors, including the Nernst coefficient, the geometry and the impedance. For the 18 μm cross-structure, we calculate the magnetization angle sensitivity to be 
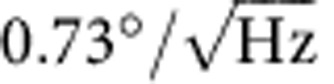
 (further information in Supplementary Note 4 and Supplementary Fig. 7). Because of its picosecond duration, the TRANE signal is sensitive to the microwave impedance of the sample, with the strongest signal occurring when the sample impedance matches the 50 Ω impedance of the measurement circuit. In addition, the electrical TRANE signal scales as *d*^2^/*w* for a probe diameter, *d*, and a channel width, *w*^21^. Thus, a TRANE signal collected with a nanoscale excitation source (<200 nm) could remain large, provided that *w* is also scaled down. For comparison, the signal scaling of magneto-optical microscopy is essentially independent of the device geometry above the optical diffraction limit of *d*∼*λ*/(2 NA) ∼200 nm for blue light.

### Spatial measurement with focused pulsed thermal gradients

**Figure 2:**
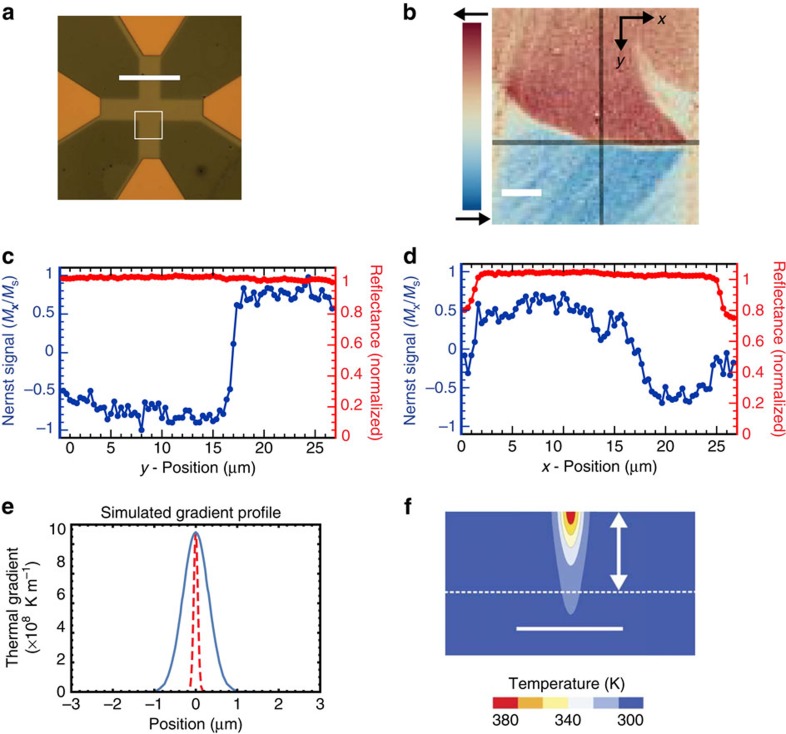
Spatial resolution of magnetic imaging using a thermal gradient. (**a**) Optical micrograph of the cobalt structure measured with TRANE. Scale bar is 50 μm, the white square outlines the region imaged with TRANE and shown in **b**. (**b**) Image of magnetic structure taken with TRANE. Scale bar is 5 μm, and dark lines are positions of the line cuts shown in **c** and **d**. (**c**,**d**) Line cuts of the image showing the TRANE signal in blue (normalized by the value at saturation, *M*_*s*_) and the simultaneously measured reflected light in red. (**c**) Line cut in *y*-direction (top to bottom). (**d**) Line cut in *x*-direction (left to right). We note that the reflected signal drops off at the edge of the cross while the TRANE signal goes to zero without edge artefacts. (**e**) Finite element, time-dependent simulation of the vertical component of the thermal gradient at peak applied power along the length of a long 20 μm permalloy wire as a function of distance from the spot centre. The blue line corresponds to a heat source with a Gaussian width of 311 nm. The dashed, red line corresponds to a hypothesized, nanothermal generation with a Gaussian width of 50 nm corresponding to a potential source from (for instance) a scanning probe. We note that, because of radial symmetry, the radial (in-plane) gradient gives no signal. (**f**) Temperature in a 2 μm wide permalloy wire and sapphire substrate calculated with a finite element, time-dependent simulation at peak applied power. The thermal source was a Gaussian with a width of 311 nm and peak power of 2.19 W. The two-dimensional plot shows a cut across the width of the wire through the middle of the heated region. The scale bar is 4 μm and the vertical arrow is 30 nm. The white dashed line indicates the sapphire substrate surface.

Next, we experimentally demonstrate that lateral thermal diffusion does not limit spatial resolution at the scale of a tightly focused laser by imaging the local magnetic moment of the cobalt cross. Scanning the laser across the sample, a map of the magnetization is created (Fig. 2a,b) in which domain walls are visible where the projected moment is zero. For the cobalt films studied here, the domain walls are 150–200 nm wide^25^, which is far below the apparatus resolution of 311±15 nm, defined as the Gaussian width measured from the reflectance (with a corresponding FWHM of 730±35 nm). We use this fact to evaluate the resolution of our TRANE microscope by fitting the spatial line cut across a magnetic domain wall (line cut in the *y*-direction) with the convolution of a step function and a Gaussian function of width, *δ*. Line cuts in both *y* and *x* are shown in Fig. 2c,d. The fit yields *δ*=326±70 nm (corresponding to a FWHM of 767±165 nm, the fitting procedure is described in the Supplementary Note 5 and Supplementary Fig. 8). These results suggest that the main limitation to the spatial resolution is the size of the thermal gradient spot.

To gain a deeper understanding of thermal diffusion in our magnetic thin-film samples, we performed time-dependent, finite element simulations of the picosecond heating dynamics. Using numerical simulations, we show in Fig. 2e (blue curve) that when the laser pulse is at its maximum, the vertical component of the thermal gradient does not spread laterally beyond the pulsed heat source. Interestingly, our simulations show this statement is true even for a hypothetical nanoscale thermal source (dashed red curve in Fig. 2e). The simulated two-dimensional temperature profile is shown in Fig. 2f.

### Stroboscopic measurement of magnetic dynamics

**Figure 3:**
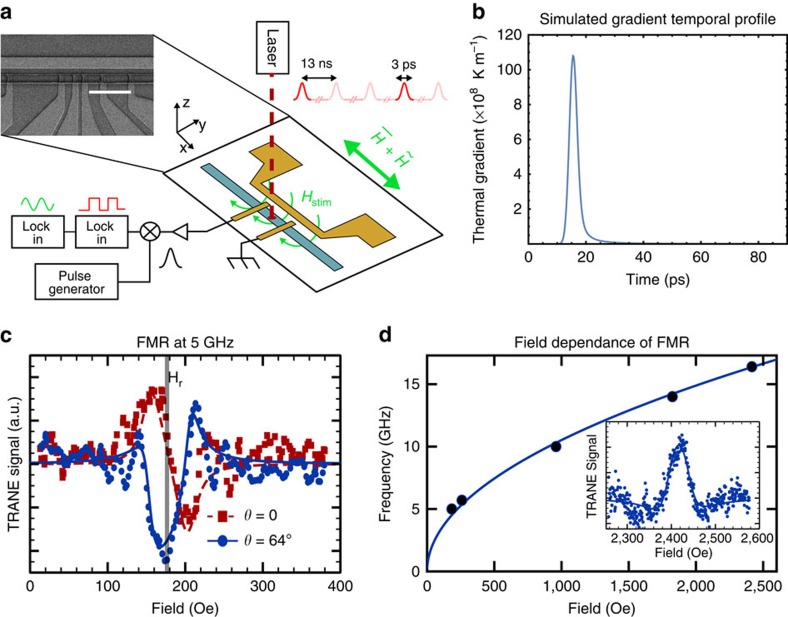
Measurement of magnetic dynamics using TRANE. (**a**) Schematic of the experimental set-up used to measure FMR in permalloy wires. The 2 μm wide permalloy wire (blue) is stimulated by the magnetic field, *H*_stim_ from a 2 μm copper wire 1 μm away from the permalloy wire. The contacts used to measure the TRANE signal were separated by 3 μm. The external field is applied parallel to the wire. 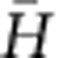 is the mean static field and 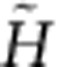 is low amplitude modulation used as a reference for the second cascaded lock-in amplifier. The white scale bar is 5 μm. (**b**) Time-dependent numerical simulation of the thermal gradient. (**c**) FMR spectra measured using a 5 GHz excitation field and probed at two different phases in the precessional cycle. Red squares show the measurement at a phase of *θ*=0° with the fit shown as the dashed red curve, blue circles show the measurement at a phase of *θ*=64° with the fit shown as the solid blue curve. The vertical gray line is a guide to the eye showing the location of the resonant field, *H_r_*, determined by the fit. (**d**) Plot of the resonant frequencies as a function of the resonant field determined by fitting. The solid line is a fit to the Kittel equation. An FMR spectrum measured at 16.4 GHz is shown in the inset. For all the FMR spectra, the points show the data after smoothing over three neighbouring points. The lines are a fit to the linear combination of symmetric and anti-symmetric Lorentzians after accounting for the modulation frequency.

We study TRANE's temporal resolution by stroboscopically measuring ferromagnetic resonance (FMR) in Ni_20_Fe_80_ (permalloy) wires using the apparatus depicted in Fig. 3a. To excite magnetization dynamics, a microwave frequency current is passed through an on-chip copper wire to generate an out-of-plane a.c. magnetic field in the permalloy. The wire axis of the sample is aligned parallel to the applied static magnetic field and the contacts are placed so as to measure the *M*_*y*_ component of the magnetization (perpendicular to both the wire axis and the excitation field). To measure *V*_TRANE_ for FMR measurements, we use an additional step of lock-in detection to reference the signal to both the optical chopping frequency and to a small amplitude field modulation along the direction of the static magnetic field. The dual referenced lock-in detection allows us to reject the contribution from a.c. currents induced by microwave driving.

In this scheme, the precessing *M*_*y*_(*t*) is stroboscopically transduced by the short thermal pulse. By choosing a microwave drive frequency, *ω*, commensurate with the laser repetition rate, we create a constant relationship between the arrival of laser pulses and the phase of the excitation field. Starting from equation (1), the anomalous Nernst voltage generated at the sample's electrical pick-ups is *V*_ANE_(*t*)=−*βN*∇*T*_*z*_(*t*)*M*_*y*_(*t*), where *β* is a proportionality constant. Because the magnetization precesses at frequency *ω*, it has a constant phase, *φ*, with respect to the laser repetition rate. Therefore, the expression simplifies to *V*_ANE_(t)≈−*β*∇*T*_*z*_(*t*)*m*_*y*,prec_ cos(*ωt*+*φ*) where *m*_*y*,prec_ is the precessional amplitude along *y*. The phase is determined by the relative delay between the laser pulses with respect to the a.c. magnetic excitation, and defines which segment of the precessional cycle is probed. The final signal we measure after the amplifiers and mixer is described by an integral over the laser pulse period, *T*, such that


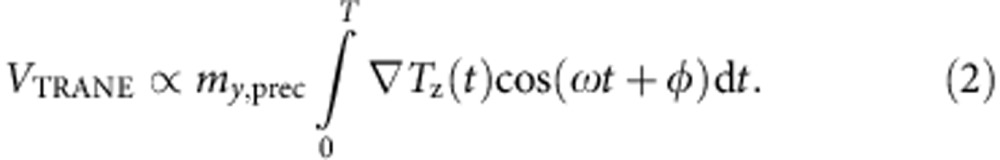


Thus, the magnetic moment is interrogated only when the temperature gradient, ∇*T*_z_(*t*), is non-zero. In our case, numerical simulations (Fig. 3b) suggest that ∇*T*_z_(*t*) is non-zero for ∼10 ps. We note that if ∇*T*_z_(*t*) persists over a duration comparable to a half period of the precession, *π*/*ω*, then *V*_TRANE_ will integrate to nearly zero. Below we will use this fact to put an upper bound on the time duration of ∇*T*_z_(*t*) by measuring FMR at increasing frequencies. A complete discussion of the stroboscopic time resolution of TRANE microscopy is given in the Supplementary Note 1.

In Fig. 3c, we plot the stroboscopic TRANE measurement as a function of magnetic field and see an FMR peak corresponding to a maximum oscillation angle. The magnetization is excited by a 5.00-GHz stimulation to a maximum angle of 0.07° at 176 Oe. The measurement sensitivity is 
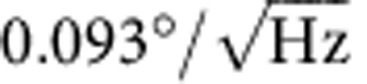
, which is improved from the cobalt cross-sample chiefly because of the reduced sample width that increases the ratio *d*^2^/*w*. Figure 3c shows two different phases of the same FMR frequency controlled by electrically shifting the time delay between the microwave magnetic field drive and the laser probe by 50 ps. The corresponding shift of the lineshape results from measuring the magnetic moment at a different part of the precessional cycle, testifying that the short-lived thermal gradient is a time-domain probe.

The FMR data are analysed by fitting to linear combinations of symmetric and anti-symmetric Lorentzian functions modified to account for the magnetic field modulation (Supplementary Note 6 and Supplementary Fig. 9). From the fits, we extract a phase difference between the two of 64°±24°. The discrepancy from our expectation of a 90° shift might be due to phase drift between the excitation and the measurement on laboratory timescales, or because our model accounts only for a single, uniform FMR mode. Close inspection of the two data sets in Fig. 3c reveals additional features that are anti-correlated between measurement phases. This suggests more complicated magnetic behaviour than we model, including the existence of additional magnetic modes that may influence the accuracy of the phase we extract from fitting. Although full imaging and analysis of these modes is a capability of TRANE microscopy, their detailed study is beyond this scope of the present demonstration.

As we increase the frequency of the magnetic excitation, we find that (as expected) the FMR resonance field is well described by the Kittel equation, 
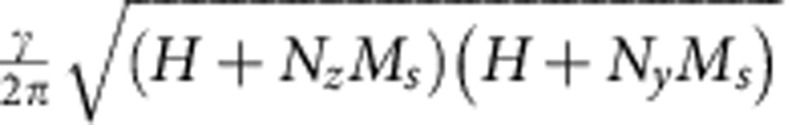
, as shown in Fig. 3d, where *γ* is the gyromagnetic ratio and *H* is the applied field. Here, we use demagnetizing factors *N*_*y*_=0.015 and *N*_*z*_=0.985, which are determined separately with measurements of the hard axis magnetic saturation. From these fits, we find an effective magnetic moment 4*πM*_*s*_=840 e.m.u. per cm^3^ and a Gilbert damping parameter, *α*=0.009±0.001. The damping in this sample is consistent with separate FMR measurements that we made by electrically monitoring the d.c. rectification voltage. These results are also in excellent agreement with literature values for permalloy^26^,^27^. The consistency among our various measurements and prior reports support the idea that the local, transient heating of the sample during measurement does not significantly alter its dynamical properties as probed by TRANE microscopy.

As mentioned above, The FMR data shown in Fig. 3d allow us to refine the upper bound of the thermal gradient lifetime. From equation (2) we see that, for *V*_TRANE_ to stroboscopically measure periodic motion, the thermal gradient decay time must be shorter than one-half the period of magnetic precession, otherwise the signal would average to nearly zero. In the experiment (inset to Fig. 3d), we observe strong FMR spectra up to 16.4 GHz (period of 60 ps), which is the highest frequency that we can produce with our microwave electronics. Therefore, we conclude that the thermal gradient must decay in <30 ps. This is supported by our time-dependent finite element modelling (Fig. 3b), which shows that the thermal gradient pulse has a width of ∼10 ps for these samples.

## Discussion

Although the thermal spot size used in this demonstration is limited by optical diffraction, TRANE microscopy is a viable strategy for high spatiotemporal resolution. The ANE interaction time and the electron thermal carrier wavelength are both short in comparison with the scales of magnetic dynamics and the spatial variation of magnetization. Because thermal gradients are not fundamentally limited by optical diffraction, microscopy based on magneto–thermal interactions has no fundamental barrier to decreasing the spatial resolution. Therefore, the spatiotemporal resolution of TRANE microscopy is predominantly limited by the generation and evolution of the localized thermal gradient. The thermal gradient diameter—and therefore the minimum resolvable feature—could be reduced below the far-field optical diffraction limit by scanning a light-confining plasmon antenna. Confined local heating using this approach has been previously demonstrated in the context of heat-assisted magnetic recording^28^,^29^.

The above results demonstrate that for the thin-film samples studied here, TRANE microscopy has temporal resolution below 30 ps and spatial resolution at the limit of focused light. Introducing this time-domain capability enables the use of TRANE for phase-sensitive dynamical microscopy in cases of uniform ferromagnetic excitation as examined here, but it could also be used to image local relaxation dynamics in a pump-probe experiment. Applying these capabilities in a table-top imaging platform with potential for nanoscale spatial resolution could enable unprecedented access to time-resolved magnetization dynamics in support of the burgeoning field of high-speed magnetoelectronics.

## Methods

### Sample preparation

For measurements of spatial resolution, 30 nm thick cobalt films were deposited by electron beam evaporation onto sapphire substrates. Photolithography and ion milling was used to pattern the films into square crosses as pictured in Fig. 2a. For the spatial map of magnetization presented, the cross-arms were 18 μm wide. Electrical contact was made by wire bonding to evaporated copper contacts.

The samples used for FMR measurements were 30 nm thick Ni_20_Fe_80_ (permalloy) films deposited by d.c. magnetron sputtering at a base pressure below 10^−7^ Torr. The films were patterned with e-beam lithography and ion milled into wires 2 μm wide and 950 μm long. Evaporated copper contacts 1 μm wide were fabricated to contact the permalloy wire with a range of separations to enable a d.c. impedance match close to 50 Ω. The contacts chosen for the measurement were 3 μm apart and had a d.c. resistance of 74 Ω. The wire we used as a microwave antenna to excite magnetization dynamics was fabricated in a lift-off process to be 2 μm wide, 50 μm long and 102 nm thick. It was positioned 1 μm away from the permalloy wire and had a d.c. resistance of 48 Ω.

### Thermal gradient generation

Local thermal gradients were generated by focusing light from a titanium:sapphire laser (Coherent MIRA 900 dual) tuned to 792 nm with 3 ps pulses and a fluence at the sample of 2.3 mJ cm^−2^. The repetition rate was controlled with an electro-optic modulator/pulse picker. We used a repetition rate of 76 MHz for measurements of the spatial imaging and 25.3 MHz for the FMR measurements. An optical chopper was used to modulate the optical pulse train at 9.7 kHz. To scan the beam, we used a 4-F optical path in combination with a voice-coil controlled fast-steering mirror. The light was focused using a 0.90 NA air objective. The entire apparatus is on a 5 by 10 foot optical table.

To determine the temperature change induced by the laser pulses, we measured the temperature increase using electrical measurements in conjunction with numerical simulations. The full temperature measurement analysis is presented in Supplementary Note 2.

### Detection

To detect the *V*_ANE_ pulses during TRANE measurement of the magnetic moment, we connect the sample voltage contact to a microwave transmission line through a coplanar waveguide soldered to a type-K connector. The signal is passed through a low-pass filter with a 4 GHz break frequency to attenuate GHz frequency artefacts from inductive electrical coupling between the copper antenna and permalloy wire. After the filter, the signal is amplified by 40 dB with a 0.1–1 GHz bandwidth. The amplified pulse train is sent to the radio frequency port of an electrical mixer, where it is mixed with a 1.5 ns pulse train from a pulse/pattern generator that is referenced to the laser repetition rate. When the two pulse trains temporally overlap on the mixer, a voltage modulated by the optical chopper (and, for FMR, the field modulation) is passed to a low-frequency preamplifier before being sent to a lock-in amplifier.

To determine the amplitude of *V*_ANE_ pulses before amplification and electrical mixing, we calibrated the transfer function of the *V*_ANE_ measurement circuit by measuring the signal produced by electrically generated reference pulses and systematically varying their widths. We find that our detection circuit transfer coefficient is 0.47±0.04 for a 10 ps signal pulse (Supplementary Note 7 and Supplementary Fig. 10). Using this calibration, we measure that the anomalous Nernst coefficient in permalloy is 2.7±0.3 × 10^−7^ V T^−1^  K^−1^, which is consistent with previous reports^20^,^21^,^22^ accounting for differences in the coefficient that arise due to variations in resistivity and thickness between samples^30^.

### Two-dimensional imaging

Imaging the static magnetic moment is performed by measuring the *V*_TRANE_ along a channel perpendicular to the applied magnetic field so that the maximum signal was obtained during saturation of the magnetic moment. The multi-domain state was prepared by saturating the cross with a 130 Oe field and decreasing the field to 32 Oe. For the data in Fig. 2b–d, we used a 250 nm step and a lock-in time constant of 500 ms.

### FMR excitation

FMR was excited in the samples using a microwave signal produced by an arbitrary waveform generator (AWG) with a clock referenced to the laser repetition rate. This clock is multiplied up within the AWG to a sampling rate of 19.98 GS s^−1^ derived from the 25.3 MHz laser pulse repetition rate. The waveforms from the AWG can be delayed in steps of 50 ps with respect to the laser pulses without re-triggering, allowing resonant behaviour of different phases to be observed. For excitation frequencies above 5.7 GHz, the output frequency of the AWG was doubled or quadrupled with electrical frequency multipliers to achieve frequencies up to 16.4 GHz. This excitation signal was then amplified to a power between 13 and 20 dBm, and coupled to the copper stimulation wire.

### TRANE detection of FMR

FMR was detected by using a second lock-in with dual demodulation. In this technique, two modulation sources at different frequencies are used. The signal is extracted by first demodulating the input referenced to the optical chopper. The resulting signal is then sent to a second demodulator (time constant of 1 s) that is referenced to a 5–10 Hz modulation of the magnetic field.

## Additional information

**How to cite this article:** Bartell, J. M. *et al*. Toward a table-top microscope for nanoscale magnetic imaging using picosecond thermal gradients. *Nat. Commun*. 6:8460 doi: 10.1038/ncomms9460 (2015).

## Supplementary Material

Supplementary Information Supplementary Figures 1-10, Supplementary Table 1, Supplementary Notes 1-7 and Supplementary References
