# Supplementary material for: Towards a table-top microscope for nanoscale magnetic imaging using picosecond thermal gradients
Source: Nat Commun. 2015 Sep 30;6:8460. doi: 10.1038/ncomms9460 (PMC4598727; doi:10.1038/ncomms9460)
Supplement: Supplementary Information — Supplementary Figures 1-10, Supplementary Table 1, Supplementary Notes 1-7 and Supplementary References [file ncomms9460-s1.pdf]

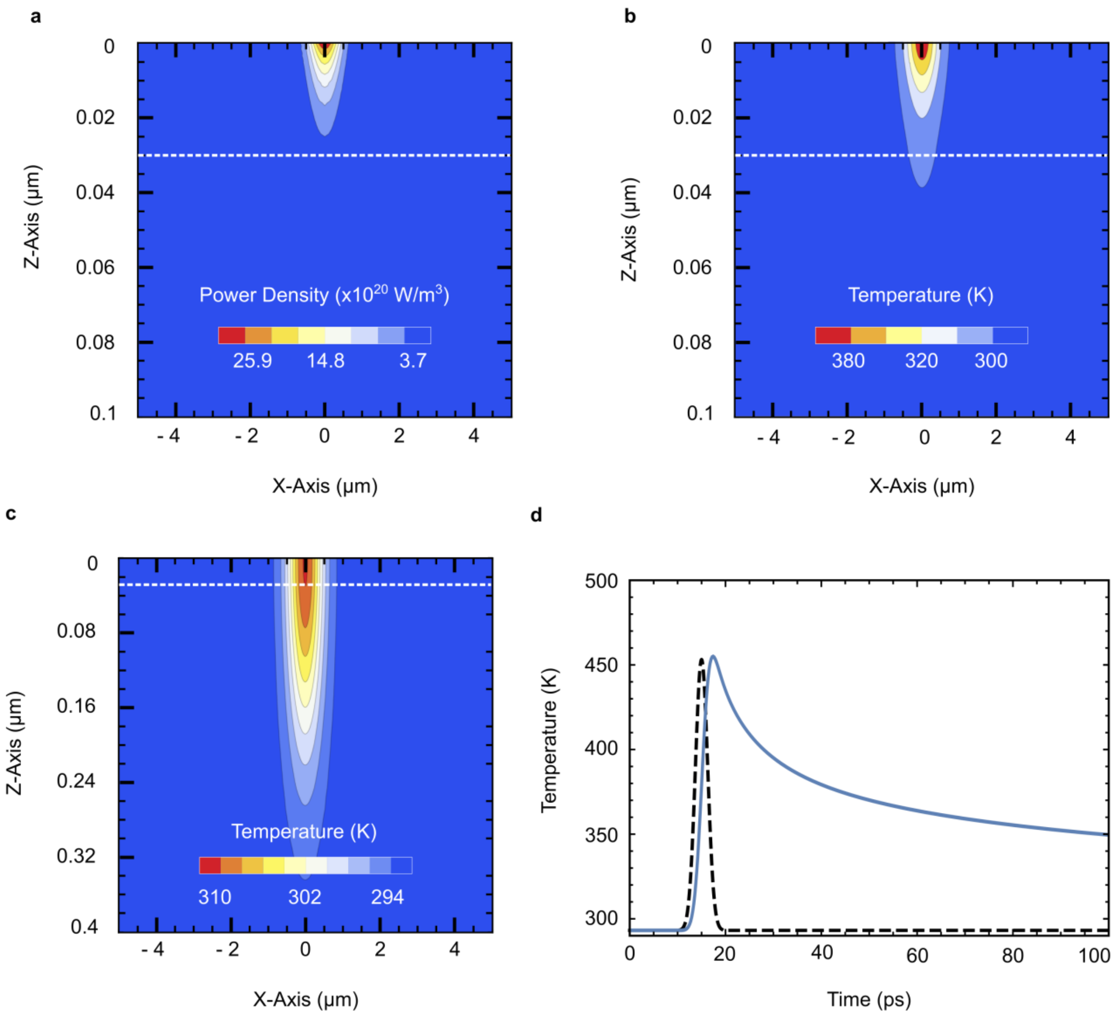

Supplementary Figure 1 | Simulated Spatial and Temporal Temperature Profiles. a, Spatial profile of the heat source, $Q(\mathbf{x},t)$, for the 311 nm spot size. **b-c,** Temperature profiles across an axial slice of the thermal source of the 311 nm Gaussian width. The dashed line indicates the interface between the permalloy wire and the sapphire. **b,** is the temperature at the peak of the pulse and **c**, is the temperature 982 ps after the peak. **d,** Time dependence of the laser induced temperature increase for 311 nm Gaussian width thermal spot. The dashed line shows the temporal profile of the heat source in arbitrary y – axis for reference.

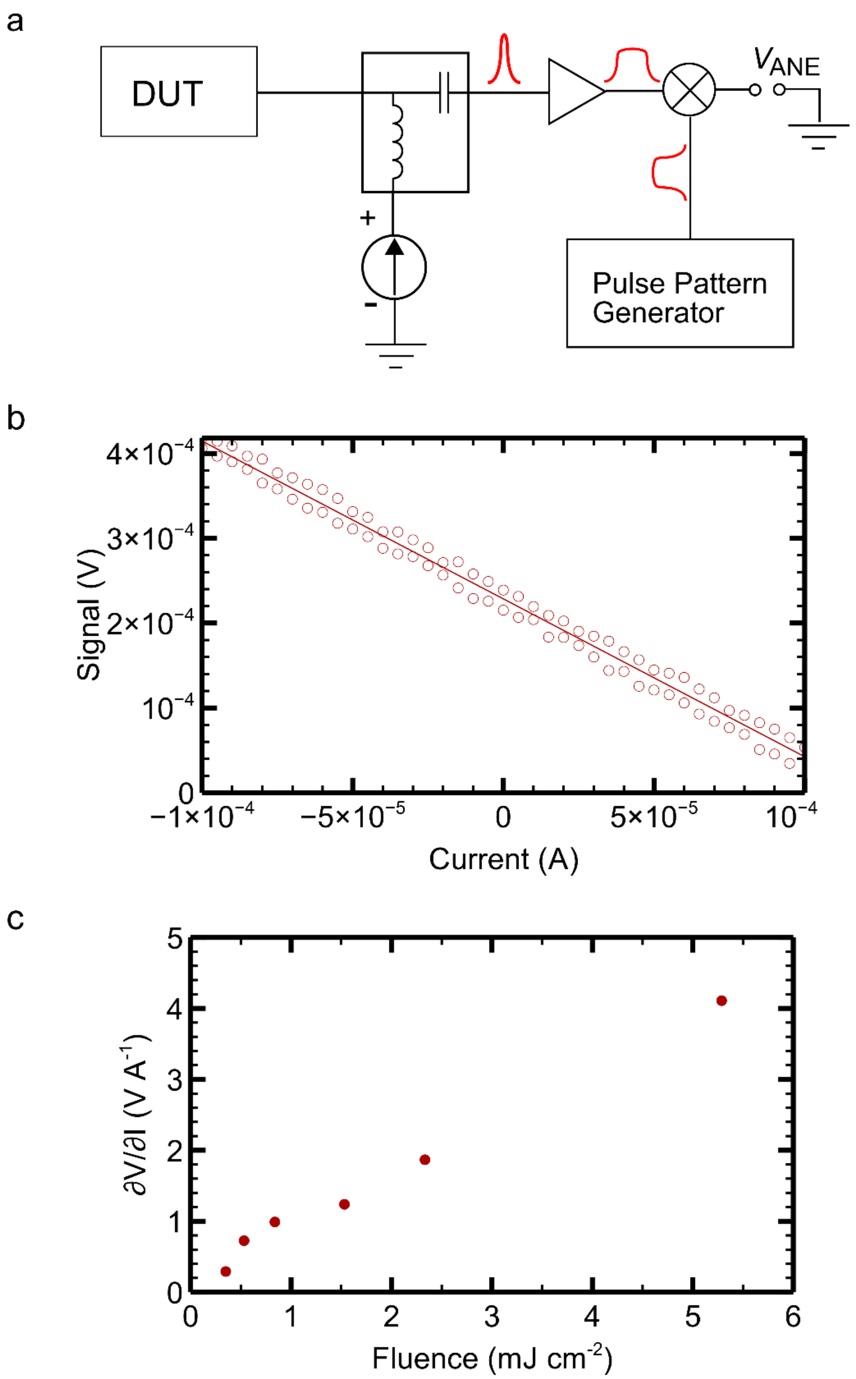

Supplementary Figure 2 | Measurement of Temperature Dependent Resistance. a, Circuit setup to measure the temperature induced resistance change. **b,** The collection signal as a function of the applied DC current to measure the resistance change due to heating from the laser pulse. **c**, The slope of the collection signal versus applied DC current for various laser powers.

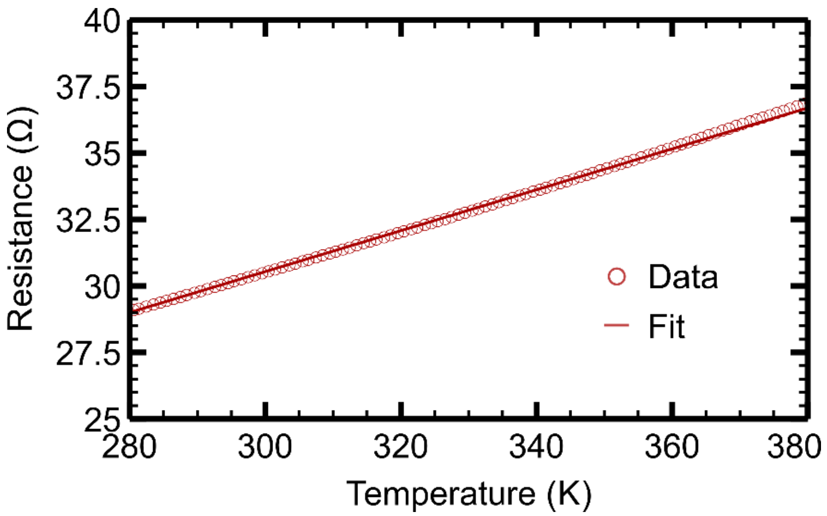

Supplementary Figure 3 | Temperature Dependent Resistivity With the PPMS, we measure the temperature dependence of the resistance of a 30 nm thick permalloy sample.

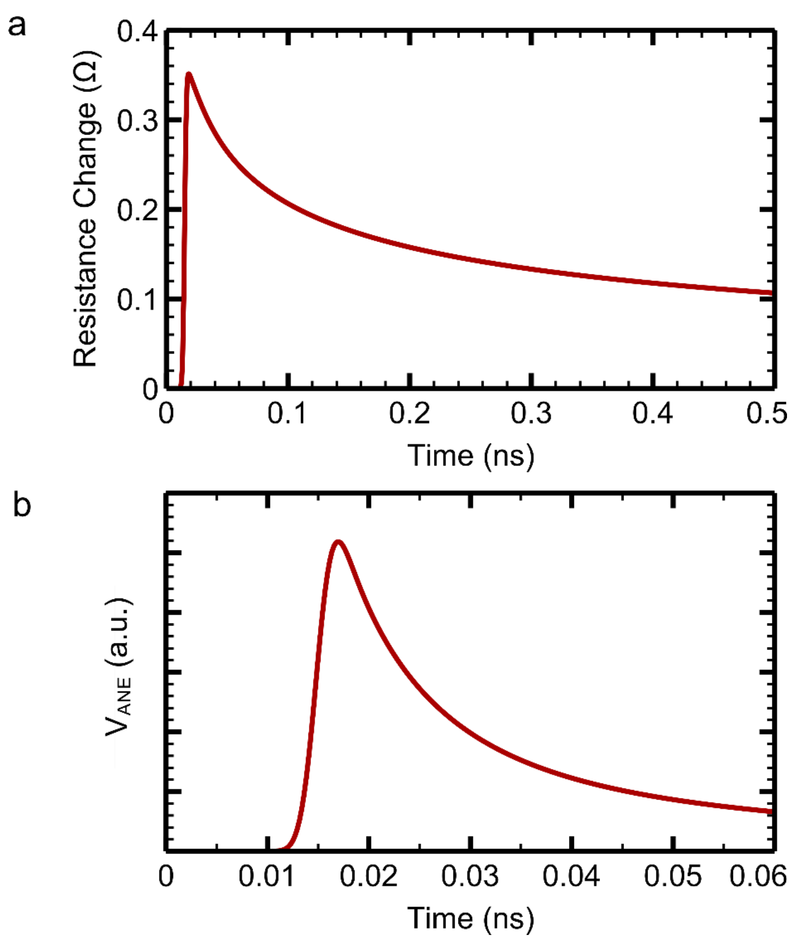

Supplementary Figure 4 | Calculated Laser Induced Resistant Change and V_{ANE} Signal. a, The total resistance change calculated from the numerically simulated temperature change from heating due to the laser. **b,** The temporal response of the anomalous Nernst voltage. These calculations correspond to a Gaussian laser pulse with its peak at 15 ps.

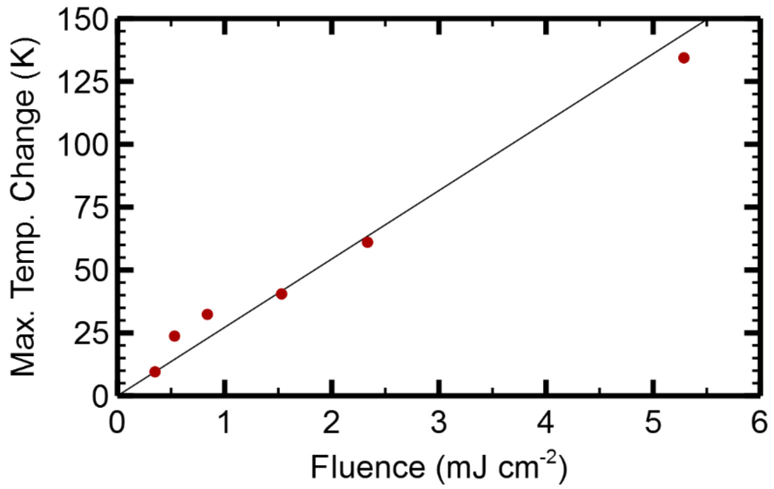

Supplementary Figure 5 | Laser Induced Temperature Change. The measured maximum temperature change due to heating from the laser as a function of the laser fluence.

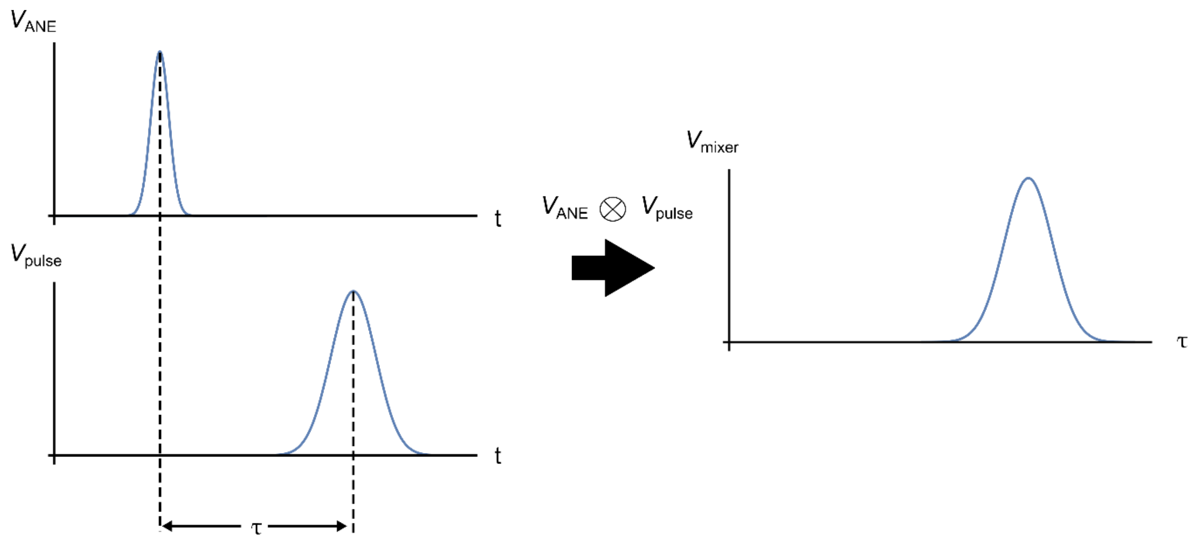

Supplementary Figure 6 | Schematic Depiction of Temporal Convolution Using an Electrical Mixer A depiction of the idealized temporal convolution measurement using a .5-18 GHz mixer. For two pulses of different widths, the resulting measurement has a pulse full width at half maximum that is equal to the width of the longest pulse.

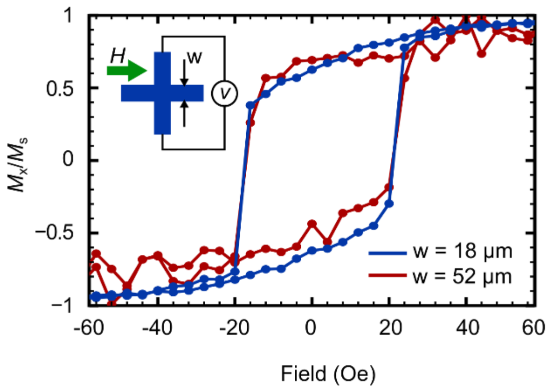

Supplementary Figure 7 | Comparison of Hysteresis Measurement Sensitivity In this graph we plot TRANE – measured hysteresis loops for two different cross sizes. We observe that the sensitivity is $\theta_{\min} = 4.6^{\circ}/\sqrt{\mathrm{Hz}}$ for the 52 μm cross and $\theta_{\min} = 0.73^{\circ}/\sqrt{\mathrm{Hz}}$ for the 18 μm wide cross.

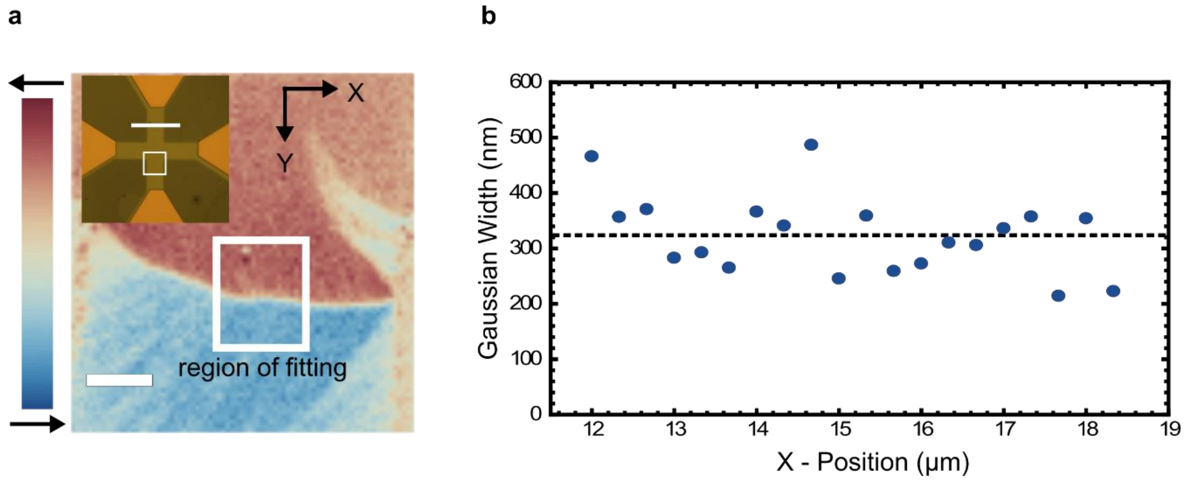

Supplementary Figure 8 | Region and Results of Spatial Fitting. a, Spatial map of the static magnetic moment showing the region used for the lateral resolution measurement. The scale bar is 5 μm. The inset shows an optical micrograph of the device studied, the scale bar is 50 μm and the white square outlines the region imaged with TRANE. **b,** Gaussian width of the pulse that was convolved with thestep determined by fitting. The x-axis is the horizontal coordinate of the line cut used and the dashed line indicates the mean of 326 nm.

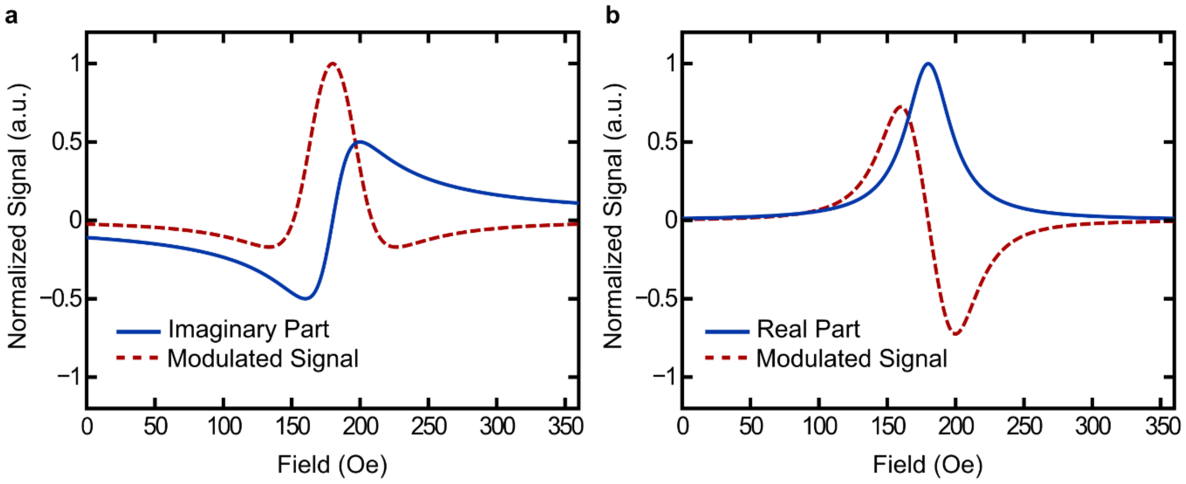

Supplementary Figure 9 | Modification of Lorentzian Functions. The blue curve in each plot shows the modeled, normalized - Lorentzian response function for the projected amplitude of the FMR precession for a resonant field, H_r = 180 Oe and line-width of 80 Oe. The dashed, red curves show the corresponding signal line shape as detected by the lock-in when using a modulation amplitude of 20 Oe.

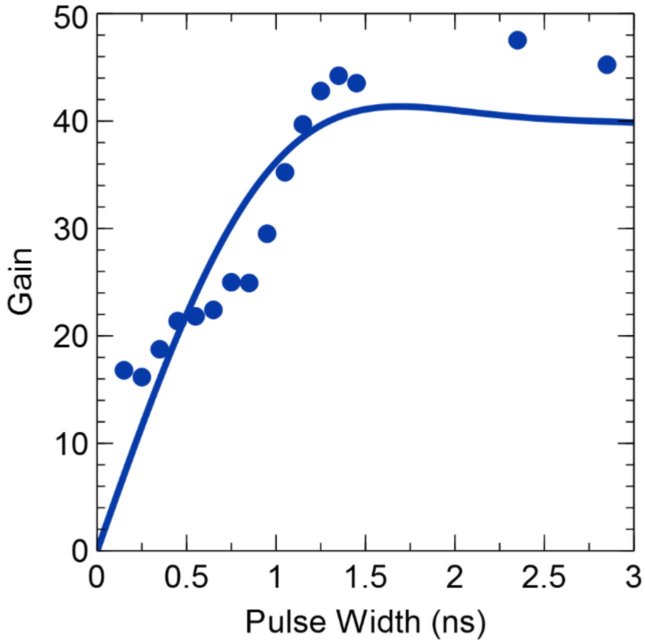

Supplementary Figure 10 | Collection Circuit Gain. We plot the dependence of the collection circuit gain on the temporal width of a calibrating square pulse. The model used to fit the data estimates a transfer coefficient of 0.47 ± 0.04 for a 10 ps ANE pulse.

Supplementary Table 1 | Material Parameters used for Simulation

Material	Thermal Conductivity (W m^{-1} K^{-1})	Specific Heat (J g^{-1} K^{-1})	Density (g cm^{-3})
Sapphire[1]	30.3	0.764	3.98
Permalloy	46.4[2]	0.43[3]	8.7[4]

Supplementary Note 1 | Independence between TRANE Temporal Resolution and Circuit Bandwidth.

This section shows how the temporal resolution of TRANE is only dependent on the lifetime of the thermal gradient, not on the bandwidth of the collection circuit. We discuss how the mixer is used to detect the pulsed signal and give relevant details pertaining to circuit bandwidth. An ideal frequency mixer outputs the voltage multiplication between two input ports. Here, we label one input the sample voltage and the second input as the reference or local oscillator. If we set the local oscillator to a fixed frequency sine wave, then the mixer acts as a homodyne detector at the local oscillator frequency, producing a DC component at the output when the input is the same frequency as the local oscillator. Similarly, the mixer can be used to detect a pulse train. In this case, instead of a sine wave for the local oscillator, we use a reference pulse train with controllable duty cycle and relative delay.

To show the mixer output from a pulse train reference, we express the voltage multiplication in terms of a Fourier series expansion. Here, we define our Fourier expansion as

$$V(t) = \sum_{k} c_n e^{i\,2\pi kt/T}, \tag{1}$$

where T is the period, t is the time, and c_n are the Fourier coefficients defined as

$$c_n = \frac{1}{T}\int_{-T/2}^{T/2} dt\, V(t) e^{-i\,2\pi kt/T}. \tag{2}$$

By applying the Fourier series expansion, the DC component of the mixer output is

$$V_{\mathrm{m}} = \frac{1}{V_0}\sum_{k=-K}^{K} c_k^{\mathrm{s}} c_{-k}^{\mathrm{r}}, \tag{3}$$

where c_k^{s} and c_{-k}^{r} are the Fourier components of the sample voltage and reference voltage respectively. We have a multiplicative factor V_0 which accounts for the amplifier circuit gain and total insertion loss. $K = f_{\mathrm{max}}/f_0$ is the cutoff factor set by the bandwidth of the electrical components, f_{max}, with f_0 as the laser repetition rate. We can see from Supplementary Equation 3 that we can maximize the output signal by setting the reference to have the same Fourier components as the pulsed signal. As expected, with a pulse train as the input signal, it is best to mix with a pulse train as a reference.

The bandwidth of the collection circuit does not affect the temporal resolution of TRANE. To show this, without loss of generality, we assume the temperature gradient is constant at the laser spot and 0 everywhere else. With this assumption, the time dependent ANE voltage V_{ANE} from the resistor model is given by

$$V_{\mathrm{ANE}}(t) = \frac{\pi r^2}{w}(N\, M_{\mathrm{s}})\nabla T_{\mathrm{z}}(t) m_{\mathrm{y}}(t). \tag{4}$$

Where N is the Nernst coefficient, w is the voltage channel width, r is the thermal gradient lateral radius, and M_{s} is the magnetic saturation. This is for measurement of the y-axis component of the magnetic moment, m_{y} with a perpendicular-to-the-plane thermal gradient, ∇T_{z}. Applying this equation to Supplementary Equation 3, the measured voltage from the collection circuit can be expressed as

$$V_{\mathrm{m}}(t) = \frac{1}{V_0}\left(\frac{\pi r^2}{w}\right)(N\, M_{\mathrm{s}}) \sum_{k=-K}^{K} c_{-k}^{\mathrm{r}} \int_{-T/2}^{T/2} dt \left(e^{i\frac{2\pi k t}{T}}\right) \nabla T_{\mathrm{z}}(t) m_{\mathrm{y}}(t).$$

5)

The temperature gradient is non-zero only for a short time t_{ANE} ($t_{\mathrm{ANE}} \approx 10$ ps according to numerical simulations). Using the fact that $t_{\mathrm{ANE}} \ll 1/f_{\mathrm{max}}$, which is true for the 1 GHz bandwidth circuit components, we can approximate the measured signal as

$$V_{\mathrm{m}}(t) \approx \left[\frac{1}{V_0}\left(\frac{\pi r^2}{w}\right)(N\, M_{\mathrm{s}})\left(\sum_{k=-K}^{K} c_{-k}^{\mathrm{r}}\right)\right]\int_0^{t_{\mathrm{ANE}}} dt\, \nabla T_{\mathrm{z}}(t) m_{\mathrm{y}}(t). \tag{6}$$

This shows that TRANE measures the magnetic moment over the time period of t_{ANE}. Therefore, the time resolution of TRANE is determined by the lifetime of the temperature gradient and it is not limited by the frequency bandwidth of the collection circuit.

Supplementary Note 2 | Determination of Laser Induced Temperature Change

The finite element modeling of the thermal gradient evolution used for determining the temperature and thermal decay times was performed using the COMSOL Multiphysics Heat Transfer Module. We consider a single temperature diffusive model in which the laser is treated only as a heat source, rather than considering different phonon and electron temperatures. This is justified by the fact that the optically excited electrons are thermalized on time scales comparable to the laser pulse width of 3 ps[5].

The spatiotemporal evolution of the thermal gradient in our system is calculated numerically with the Fourier diffusion equation using the material parameters given in Supplementary Table 1. The heat source $Q(\mathbf{x}, t)$, is given by,

$$Q(\mathbf{x}, t) = \frac{Q_o}{2\,\pi\,\delta_{\mathrm{x}}\,\delta_{\mathrm{y}}\,d}\, e^{-\frac{x^2}{2\,\delta_{\mathrm{x}}^2}-\frac{y^2}{2\,\delta_{\mathrm{y}}^2}} e^{-\frac{z}{d}}\, e^{-\frac{t^2}{2\tau^2}} \tag{7}$$

where, δ_{x} and δ_{y} are the Gaussian widths in the x and y direction of the laser spot (311 nm), d is the skin depth (12 nm), Q_{o} is the incident peak power of a single pulse (2.19 W), and τ is the pulse Gaussian temporal width of the 3 ps pulse.

The results of the simulations yield spatiotemporal profiles of the temperature and thermal gradient shown in Supplementary Figure 1. To apply the simulation for quantitative analysis we need a sample specific scaling factor determined experimentally (See below).

When the laser induces a temperature increase to create the TRANE signal, it also creates an increase in the local resistance. If there is electrical current in the sample, the resistance change creates an additional voltage contribution that is independent of the sample magnetization. In this section, we use the signal from the local resistance change to measure the temperature profile of the sample due to heating from the laser. We determine the local resistance change by measuring the signal dependence on an applied DC electrical current. The DC current is applied to the sample by introducing a bias-tee into the circuit as shown in Supplementary Figure 2a. To show the relationship between the resistance change and the measured voltage, we begin with the sample voltage, which is given by

$$V_{\mathrm{sample}}(t) = V_{\mathrm{ANE}}(t) + I(t)\,R(t), \tag{8}$$

where V_{ANE} is the voltage from the anomalous Nernst effect and $I(t)\,R(t)$ is the Ohmic voltage. For the current scenario, we set a constant applied current, while the resistance and V_{ANE} vary in time. From Supplementary Note 1, we find the voltage at the mixer output is given by

$$V_{\mathrm{m}} = \frac{1}{V_0} \sum_{k=-K}^{K} c_{-k}^{\mathrm{r}} \left[c_n^{\mathrm{ANE}} + \int_{-\frac{T}{2}}^{\frac{T}{2}} dt\, I_{\mathrm{DC}} R(t) e^{-\frac{i2\pi kt}{T}} \right]. \tag{9}$$

By chopping laser power, the lock-in voltage from the mixer signal is given by

$$V_{\mathrm{LI}} = \frac{1}{V_0} \sum_{k=-K}^{K} c_{-k}^{\mathrm{r}} \left[c_n^{\mathrm{ANE}} + I_{DC}\, c_k^{\Delta\,\mathrm{R}} \right], \tag{10}$$

where $c_n^{\Delta \mathrm{R}} = \int_0^T dt\, \Delta R(t) e^{-i\,(2\pi k)t/T}$ is Fourier series component of the change in the resistance, ΔR, from room temperature. We show the results of these measurements in Supplementary Figure 2b, which displays the expected linear relationship between the collection signal and the applied DC current. We repeat the measurements at various laser powers and plot the slope as a function of laser power in Supplementary Figure 2c. These measurements were performed in the presence of a large saturating magnetic field, so that we can neglect current induced magnetization effects that may change the anomalous Nernst signal. Therefore, by relating the resistance change to a temperature increase, we can quantitatively determine the heating induced by the laser.

It is non-trivial to directly convert this data into a measure of resistance due to the non-linearity of the circuit components. Instead, we compare this data to numerical simulations and calculations. We numerically simulate the temperature profile to calculate its corresponding resistance change and the resulting collection signal. Due to the unknown absorption coefficient of the sample, there is an uncertainty in the absolute value of the temperature change. Therefore there will be an overall factor which is determined by comparing the simulation results with the measured collection signal. By comparing the slopes of the calculated and measured signals as a function of DC current, we obtain the total temperature change and the temperature gradient.

We measure the temperature dependence of resistivity to map the simulated temperature profile to a total resistance change. We consider the linear response regime of the resistivity dependence on temperature, such that

$$\rho(T) = \rho_0[1 + \alpha(T - T_0)] = \rho_0[1 + \alpha\Delta T], \tag{11}$$

where ρ_0 is the resistivity at the base temperature T_0, which we set as room temperature at 293 K, and α is the temperature coefficient of resistivity. To determine the temperature coefficient of resistivity, we measure the 4-point resistance as a function of temperature with a physical property measurement system (PPMS), with the results shown in Supplementary Figure 3. With the 4-point resistance measurement, we remove contributions due to contact resistance, therefore the resistivity is related to the resistance by $\rho = \frac{RA}{L}$, where A is the cross-sectional area and L is the length between the measurement probes. By fitting the data in Supplemental Figure 3, we find the temperature coefficient of resistance in the 30 nm permalloy to be $\alpha = 0.0025\ \Omega\ \mathrm{K}^{-1}$.

We calculate the total time-varying resistance induced from laser heating by mapping the numerically simulated temperature profile to a resistivity profile using the measured resistivity versus temperature. The total resistance of the sample in terms of the spatially dependent resistivity is given by

$$R(t) = \left[\int dy\, dz \left[\int dx\, \rho\big(T(\mathbf{x}, t)\big)\right]^{-1}\right]^{-1}, \tag{12}$$

where $\rho\big(T(\mathbf{x}, t)\big)$ is the temperature dependent resistivity. Applying the linear temperature dependence of the resistivity gives

$$R(t) = \left[\int dy\, dz \left[\int dx\, \rho_0(1 + \alpha\, \Delta T(\mathbf{x}, t))\right]^{-1}\right]^{-1}. \tag{13}$$

From the measurements of the resistivity temperature dependence, it is safe to assume $\alpha\Delta T \ll R_0$, where R_0 is the resistance at T_0. Therefore, we find the total resistance change by performing a series expansion and taking the first order term to be

$$\Delta R(t) = R_0 \int^{V} \frac{d\vec{x}}{V} \alpha \Delta T(\vec{x}, t). \tag{14}$$

This shows that the total resistance change is proportional to the mean temperature change through the length of the wire. Supplementary Figure 4 shows the calculated total resistance change as a function of time for the simulated temporal profile from Supplemental Figure 1.

We note from Supplementary Figure 4 that the decay lifetime for resistance change is much larger than the pulsed anomalous Nernst voltage. This is because the anomalous Nernst voltage is dependent on the vertical component of the temperature gradient while the resistance change is dependent on the overall mean temperature. Therefore, lateral thermal diffusion from the heat source into other regions of the ferromagnet will reduce the vertical thermal gradient, causing the decay in the anomalous Nernst voltage. Conversely, lateral thermal diffusion has less influence on the overall mean temperature, and thus the total resistance changes more slowly.

We determine the temperature profile by scaling the simulated temperature profile in Supplementary Figure 1 to match the measured signal slope in Supplementary Figure 3. This matching is done by taking into account the bandwidth and the collection circuit transfer coefficient as described in Supplementary Section 7. For the typical laser fluence used of 2.3 mJ cm^{-2}, we determine a dimensionless scaling factor of 0.4. Physically, this scaling factor accounts for the unknown optical absorption coefficient. Using this factor we calculate a resulting maximum temperature increase of 60 K occurring at the surface and a corresponding maximal gradient at the surface of 4.0×10^{8} K m^{-1}. This is ~100 times greater that the value of $\sim1\times10^{6}$ K m^{-1} quoted in reference 21 of the main text for a CW laser[6]. This highlights the distinction between pulsed and CW laser measurements. We show the maximum temperature increase for various laser fluences in Supplementary Figure 5. The linearity of temperature increase as a function of laser fluence suggests that the heating from the laser is in the linear response regime.

By comparing the measured hard-axis TRANE hysteresis with the resistor model, we measure an anomalous Nernst coefficient of $2.7 \pm 0.3 \times 10^{-7}$ V K^{-1} T^{-1}. This value within an order of magnitude as reported in the literature[6-8]. There is no consensus value because the anomalous Hall coefficient for permalloy, which is related to the anomalous Nernst effect through the Seebeck coefficient, is highly dependent on the thickness and resistivity[9]. This suggests that TRANE is a viable technique to measure the anomalous Nernst coefficient in materials without specialized thermal measurement apparatus. The error for the anomalous Nernst coefficient accounts for the experimental error in the transfer coefficient and the timing of the mixing pulse. It has ignored the uncertainty of values used in the numerical simulation, which include the laser pulse temporal profile and the material parameters. These errors would change the overall scaling factor used to predict the temperature and anomalous Nernst coefficients, but it does not influence the technique to measure possible variations within the sample.

Supplementary Note 3 | Temporal Convolution Using an Electrical Mixer

For TRANE microscopy to be a truly stroboscopic, time-domain method, the voltage induced by the thermal gradient has to decay faster than the probed dynamical behavior because the thermal gradient lifetime defines the interaction time between the magnetization and the probe. Direct measurement of the V_{ANE} voltage pulse using an oscilloscope is difficult due to the short temporal duration (ps scale) and the small voltage amplitude (nV scale). As an alternative, we measure the convolution between the V_{ANE} pulse and a reference electrical pulse of known width. In this scheme, we amplify the pulse with two 10 kHz-15 GHz, 15 dB amplifiers (Picosecond Pulse Labs model 5867) and use a high-speed (.5-18 GHz) electrical mixer (Remec model MM94PG-40) to multiply the pulse, V_{ANE}, with an electrical mixing pulse, V_{pulse}. When

the relative delay between the pulses, τ, is systematically varied, we measure the temporal convolution of the two pulses, $\int_0^T V_{\mathrm{ANE}}(t)V_{\mathrm{pulse}}(\tau - t)dt$, where T is the laser pulse repetition period. The width of resulting mixed-down signal as a function of τ is most strongly determined by the width of the longest pulse entering the mixer. This measurement is depicted schematically in Supplementary Figure 6.

We note that when used for TRANE measurements, the delay τ is fixed and the pulse V_{ANE} is multiplied by a pulse from a pulse pattern generator (pulse width ~1.5 ns) after amplification by two 0.1-1 GHz bandwidth 20 dB gain amplifiers (Mini-Circuits model ZFL-1000LN+). This form of homodyne detection is used to convert the short-lived TRANE pulse into a low-frequency signal that can be measured by the lock-in. Lower frequency detection circuitry does not reduce the temporal resolution (see section 1) and allows us to filter gigahertz frequency noise induced in the magnetic channel by the AC driving field. Additionally, using a wider V_{pulse} decreases the measurement's sensitivity to variations in the delay between the V_{ANE} and V_{pulse}.

Supplementary Note 4 | Sensitivity

The sensitivity is calculated using the field-dependent magnetization measurements shown in Fig. 1c in the main text and Supplementary Figure 7. This measurement is done in the transverse geometry – the saturated moment is perpendicular to the voltage pick-ups – so that $(V_{\mathrm{TRANE}}^{\mathrm{max}} - V_{\mathrm{TRANE}}^{\mathrm{min}})$ corresponds to a 180° rotation. The standard deviation of points at saturation is taken as the detected voltage uncertainty, δ_{TRANE}. As a longer sampling time will reduce the value of δ_{TRANE} regardless of the sample, it is desirable to have a sensitivity figure of merit independent of the sampling time. Thus, the signal-to-noise ratio must be scaled to account for

the measurement rate, in the case of a lock-in measurement this is the time constant. This yields an equation for the minimum detectable angle, θ_{min}, with respect to the angle of highest sensitivity, $\theta_{\mathrm{o}} = 90^{\mathrm{o}}$, measurable with the TRANE technique.

$$\theta_{\mathrm{min}} = \frac{\delta_{\mathrm{TRANE}}}{\sin(\theta_{\mathrm{o}})\left(V_{\mathrm{TRANE}}^{\mathrm{max}} - V_{\mathrm{TRANE}}^{\mathrm{min}}\right)/2}\sqrt{TC} \quad (15)$$

Supplementary Note 5| Fitting Lateral Resolution

We measure the value for the lateral resolution by taking vertical line cuts of the 2D scan across a portion of the domain wall (Fig. 2b in the main text and Supplementary Figure 8a). The 4 μm region of the domain wall used for fitting is shown boxed in Supplementary Figure 8a. This region was chosen because it was the portion of the image with the clearest step function behavior. Fits of the line scans where done using a least squares method to find the Gaussian width, δ, amplitude, a, and center, μ, of a function derived by convolution of a Gaussian with a -1 to 1 step function. Here we define the Gaussian to be $ae^{-(x-\mu)^2/2\delta^2}$. The results of the individual fits are shown in Supplementary Figure 8b. The mean of the fits is 326 nm with a standard deviation of 70 nm, the standard deviation is used as the uncertainty as it was larger than the uncertainty of the individual fits.

Supplementary Note 6 | Modification of the Resonant Line-shapes due to Field Modulation

To measure the FMR of the permalloy wires we detect the projected magnetic moment perpendicular to the wire. The magnetic moment of the wire precesses about the externally applied magnetic field when driven by a microwave field generated in a microwave antenna patterned parallel to the magnetic wire. The FMR precession angle of a ferromagnet in the linear

response regime can be modeled as a driven damped oscillator. The projection amplitude of this motion is the linear combination of even and odd Lorentzian functions given by

$$\sin(\varphi)\frac{\frac{H-H_{\mathrm{r}}}{\vartheta}}{1+\frac{(H-H_{\mathrm{r}})^2}{\vartheta^2}} + \cos(\varphi)\frac{1}{1+\frac{(H-H_{\mathrm{r}})^2}{\vartheta^2}}, \tag{16}$$

where φ is the phase between the even and odd Lorentzian functions, H is the applied field and H_{r} is the resonant field, and ϑ is the linewidth of the Lorentzian. When measuring magnetic dynamics with TRANE, a time-varying magnetic field is applied across the ferromagnet. This induces an electrical current in the ferromagnetic wire, which creates a large background voltage across the wire which is removed with a lowpass filter.

In addition to the magnetic signal due to FMR, we also detect an induced electrical response from coupling between the microwave antenna and the magnetic channel that is detected because of the temperature induced resistance change. However, since the resistance change contribution is independent of magnetization, we are able to remove it by using a cascaded lock-in technique. We detect the signal by using two lock in amplifiers connected in series, the first demodulation was referenced to a square modulated 9.7 kHz signal from an optical chopper and the second demodulation was referenced to a 14 Oe sinusoidal field, H_{mod}, modulated at ω_{m}=10 Hz (5 Hz for FMR frequencies above 10 GHz). The TRANE signal detected by the second lock-in can be modeled by

$$\sin(\varphi)\int \frac{\frac{H + H_{\mathrm{mod}}\cos(\omega_{\mathrm{m}}t) - H_r}{\vartheta}}{1 + \frac{(H + H_{\mathrm{mod}}\cos(\omega_{\mathrm{m}}t) - H_r)^2}{\vartheta^2}} * \cos(\omega_{\mathrm{m}}t)dt$$

$$+\ \cos(\varphi)\int \frac{1}{1 + \frac{(H + H_{\mathrm{mod}}\cos(\omega_{\mathrm{m}}t) - H_{\mathrm{r}})^2}{\vartheta^2}} * \cos(\omega_{\mathrm{m}}t)\, dt. \tag{17}$$

The resulting analytical equation is then used to fit the resonance data obtained with TRANE to quantify the values of the linewidth, amplitude, phase, and center frequency. We note that the modification to the Lorentzian shape does not add free parameters to the fitting function because the modulation amplitude is a known value. The modulation does impact the uncertainty and it reduces the overall signal amplitude, but at the benefit of increased angular sensitivity.

Supplementary Note 7 | Collection Circuit Transfer Coefficient

To determine the transfer coefficient of the collection circuit depicted in Fig. 1b and Fig. 3a in the main text, we measure the collection voltage from a calibration pulse. Numerical simulations indicate that ∇T_{Z} has a width of 10 ps which for our magnetic system translates into a V_{ANE} pulse that also has a width of 10 ps. With the electronics available, we cannot create a 10 ps pulse to directly measure the transfer coefficient. Instead, we extrapolate it through measuring the gain of square pulses of wider widths. Supplementary Figure 10 shows the total gain in the collection circuit as a function of the square pulse width and the fit with our model.

We use Supplementary Equation 3 to model the gain where V_0 is the free parameter to fit the model. The pulse pattern generator signal into the mixer is treated as a periodic triangular function such that

$$V_{\mathrm{PPG}}(t) = \sum_n V_{\mathrm{PPG}}^0 \,\mathrm{Tri}\left(\frac{t + nT}{\delta}\right), \tag{18}$$

where V_{PPG}^{0} is the peak voltage, δ is the rise and fall time, $f_0 = 1/T$ is the laser repetition rate and Tri is a triangular function given by

$$\mathrm{Tri}(x) = \begin{cases} 1 - |x|, & \text{if } |x| < 1 \\ 0, & \text{else} \end{cases}. \tag{19}$$

For all measurements, we set the pulse pattern generator to have a peak voltage of 800 mV and a rise and fall time of 800 ps. We can express this in terms of a Fourier series as

$$V_{\mathrm{PPG}}(t) = \sum_{k} V_{\mathrm{PPG}}^{0}(\delta f_0)\, \mathrm{sinc}^2(\pi f_0 k \delta) e^{i2\pi f_0 k t}. \tag{20}$$

Similarly, we can express the calibrating square pulse train generated by the AWG as

$$V_{\mathrm{sq}}(t) = \sum_{n} V_{\mathrm{sq}}^{0}\, \mathrm{Sq}\left(\frac{t + nT}{\tau}\right), \tag{21}$$

where V_{sq}^{0} is the peak voltage, τ is the square wave width and Sq is a square pulse function given by

$$\mathrm{Sq}(x) = \begin{cases} 1, & if\ |x| < 1/2 \\ 0, & if\ |x| > 1/2 \end{cases}. \tag{22}$$

The square pulse train can be expressed in terms of the Fourier series as

$$V_{\mathrm{sq}}(t) = \sum_{k} V_{\mathrm{sq}}^{0}(f_0 \tau)\, \mathrm{sinc}(\pi f_0 k \tau) e_0^{i2\pi f_0 k t}. \tag{23}$$

The square pulse voltage is measured with a sampling oscilloscope to be 2.22 mV and the pulse width is varied from 300 ps to 3 ns.

With the two input signals, the DC component of the mixer output voltage is

$$V_{\mathrm{m}}^{\mathrm{DC}} = \frac{V_{\mathrm{sq}}^{0} V_{\mathrm{PPG}}^{0}}{V_0} (f_0^2 \tau \delta) \sum_{k=-K_{\max}}^{K_{\max}} \mathrm{sinc}(\pi f_0 k \tau) \mathrm{sinc}^2(\pi f_0 k \delta). \tag{24}$$

The bandwidth of the amplifiers and mixer set the maximum frequency of the sum to $K = f_0/f_{\max}$ where $f_{\max}$ is the maximum frequency bandwidth. For the measurements, the collection circuit bandwidth is limited to a maximum of $f_{\max} = 1$ GHz and the laser repetition rate is $f_0 = 25.3$ MHz. By fitting Supplementary Equation 24 to the calibration measurement with V_0 as the only free parameter, we obtain a best fit of $V_0 = 0.41 \pm 0.04$ mV.

It is desirable to describe the total measured voltage in terms of the peak anomalous Nernst voltage in terms of a collection circuit transfer coefficient. We define the transfer coefficient γ as

$$V_{\mathrm{m}}^{\mathrm{DC}} = \gamma\ \mathrm{Max}[V_{\mathrm{ANE}}(t)]. \tag{25}$$

We can determine γ by using the numerically simulated anomalous Nernst voltage in Supplementary Note 2 and applying it to calibration fit. Using the voltage multiplier coefficient from the fit in Supplementary Figure 10, our model estimates a transfer coefficient of 0.47 ± 0.04 for the numerically simulated pulse of approximately 10 ps in width.

Supplementary References

1. Dobrovinskaya, E. R. E., Lytvynov, L. L. A. & Pishchik, V. in *Sapphire: material, manufacturing, applications*. Ch. 2.(Springer, 2009).

2. Ho, C., Ackerman, M., Wu, K., Oh, S. & Havill, T. Thermal conductivity of ten selected binary alloy systems. *J. Phys. Chem. Ref. Data* **7,** 959 (1978).

3. Bonnenberg, D., Hempel, K. A., Wijn, H.P.J.: *1.2.1.2.10 Thermomagnetic properties, thermal expansion coefficient, specific heat, Debye temperature, thermal conductivity*. (ed. Wijn, H.P.J.) SpringerMaterials - The Landolt-Börnstein Database

4. Owen, E. a, Yates, E. L. & Sully, A. H. An X-ray investigation of pure iron-nickel alloys. Part 5: the variation of thermal expansion with composition. *Proc. Phys. Soc.* **49,** 323–325 (2002).

5. Eesley, G. L. Generation of nonequilibrium electron and lattice temperatures in copper by picosecond laser pulses. *Phys. Rev. B* **33,** 2144–2151 (1986).

6. Weiler, M. *et al.* Local Charge and Spin Currents in Magnetothermal Landscapes. *Phys. Rev. Lett.* **108,** 106602 (2012).

7. Von Bieren, A., Brandl, F., Grundler, D. & Ansermet, J.-P. Space- and time-resolved Seebeck and Nernst voltages in laser-heated permalloy/gold microstructures. *Appl. Phys. Lett.* **102,** 052408 (2013).

8. Slachter, A., Bakker, F. L. & van Wees, B. J. Anomalous Nernst and anisotropic magnetoresistive heating in a lateral spin valve. *Phys. Rev. B* **84,** 020412 (2011).

9. Zhang, Y. Q. *et al.* Anomalous Hall effect in epitaxial permalloy thin films. *J. Appl. Phys.* **114,** 163714 (2013).
